# Regulation of ciliary homeostasis by intraflagellar transport-independent kinesins

**DOI:** 10.1038/s41419-024-06428-9

**Published:** 2024-01-13

**Authors:** Lin Li, Jie Ran

**Affiliations:** Center for Cell Structure and Function, Shandong Provincial Key Laboratory of Animal Resistance Biology, College of Life Sciences, Shandong Normal University, Jinan, 250014 China

**Keywords:** Cell growth, Cell-cycle exit

## Abstract

Cilia are highly conserved eukaryotic organelles that protrude from the cell surface and are involved in sensory perception, motility, and signaling. Their proper assembly and function rely on the bidirectional intraflagellar transport (IFT) system, which involves motor proteins, including antegrade kinesins and retrograde dynein. Although the role of IFT-mediated transport in cilia has been extensively studied, recent research has highlighted the contribution of IFT-independent kinesins in ciliary processes. The coordinated activities and interplay between IFT kinesins and IFT-independent kinesins are crucial for maintaining ciliary homeostasis. In this comprehensive review, we aim to delve into the specific contributions and mechanisms of action of the IFT-independent kinesins in cilia. By shedding light on their involvement, we hope to gain a more holistic perspective on ciliogenesis and ciliopathies.

## Facts

- Cilium assembly involves a specialized protein transport mechanism known as intraflagellar transport (IFT), which is characterized by the bidirectional trafficking of a large protein complex along the microtubules within cilia.
- The anterograde movement of the IFT is facilitated by members of the kinesin-2 family, typically referred to as IFT-dependent kinesins.
- IFT-independent kinesins, also termed non-IFT kinesins, refer to a broad category of motor proteins that do not directly transport cargos in conjunction with the IFT system.
- Non-IFT kinesins have been found located at the basal body or axoneme of cilia and contribute to the maintenance of cilia and ciliary signaling pathways.
- Mutants of numerous non-IFT kinesins are intricately linked with a spectrum of ciliopathies.

## Open questions

- What are the specific mechanisms by which non-IFT kinesins coordinate their actions during various stages of ciliogenesis, and how do they contribute to this complex cellular process?
- What is the physiological and pathological significance of the non-IFT kinesin-mediated ciliary homeostasis in tissue development and human disease?
- While certain correlations between non-IFT kinesin and ciliopathies have been established, the underlying mechanisms remain elusive. Can non-IFT kinesins be therapeutically targeted for the treatment of ciliopathies?

## Introduction

The cilium, a highly conserved organelle, extends from the cell surface and serves a variety of functions. Structurally, it consists of the ciliary membrane, axoneme, and basal body [1, 2]. As an essential organelle, the cilium is involved in several cellular process, including sensory perception, cellular motility, signaling and communication, cell division and differentiation, and cell-to-cell communication [3]. Additionally, the cilium also contributes to tissue homeostasis and developmental signaling [4–7]. Consequently, aberrations in the structural integrity or functional capacity of cilia are implicated in a spectrum of genetic disorders collectively termed ciliopathies [4, 8]. These conditions present a diverse array of pathologies. Polycystic kidney disease (PKD), for instance, emerges from genetic mutations that trigger the formation of multiple cysts in kidney tissues [9]. Similarly, Bardet-Biedl syndrome (BBS) originates from genetic anomalies and is characterized by a multi-systemic impact. Individuals with BBS often exhibit a combination of symptoms such as progressive vision loss, obesity, polydactyly, and kidney irregularities [10–12].

Ciliary dysfunction and ciliopathies occur due to the absence or malfunctioning of proteins essential for ciliogenesis [9, 13]. These proteins required for ciliogenesis are almost synthesized in the cytoplasm and subsequently transported to the cilium through a specialized process known as intraflagellar transport (IFT) [14]. IFT is a complex and highly regulated microtubule-based transport system that facilitates the movement of proteins along the ciliary axoneme [15]. This system is anchored by two principal protein complexes: the retrograde IFT-A complex and the antegrade IFT-B complex. The bidirectional movement of IFT complexes within cilia relies on distinct motor proteins. Kinesin-2 is responsible for anterograde transport, moving the IFT-B complex toward the ciliary tip, while dynein-2 facilitates retrograde transport, returning the IFT-A complex to the base. This arrangement ensures coordinated bidirectional trafficking along the cilium [16]. The core IFT machinery, together with the motor proteins, mediate the trafficking of cilia structural and signaling proteins.

Furthermore, Recent studies have demonstrated that IFT-independent kinesins, also termed as non-IFT kinesins, which do not directly transport cargos in conjunction with the IFT system, also play important roles in ciliogenesis. For example, mutations in *Kif7*, *Kif9*, *Kif11*, or *Kif19A* causes abnormality in ciliary length as well as ciliopathy-related phenotypes [17–22]. Hence, in this review, we mainly focus on the role and mechanisms of non-IFT kinesins in ciliary formation and highlight their unique features compared to IFT kinesins. By gaining a deeper understanding of these mechanisms, insights can be gained into the modulation of ciliogenesis and can inform the development of new therapeutic strategies for ciliopathies.

## Cilia: conserved and multifunctional organelles

**Fig. 1:**
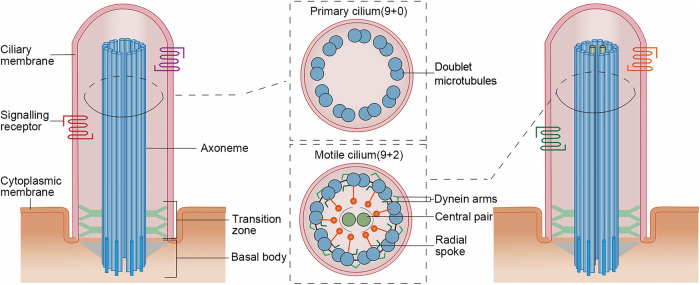
Structures of motile and primary cilia. Cilia typically have three main components: the ciliary membrane, the axoneme, and the basal body. The ciliary membrane contains numerous signaling receptors, endowing cilia with signaling functions. Axonemes are composed of doublets arranged in a “9 + 0” pattern in primary cilia. In motile cilia, the axonemes are arranged in a “9 + 2” pattern and typically include additional structures such as the central pair, radial spokes, and axonemal dynein arms, which are essential for ciliary motility. Situated between the axoneme and the basal body is the transition zone, which are connected to the ciliary membrane via Y-shaped structures.

Cilia are microtubule-based organelles prevalent in a myriad of cell types, playing vital roles in various cellular activities. These organelles can be bifurcated into two main categories: motile cilia and primary cilia. Both types feature an axoneme composed of microtubules. Motile cilia are characterized by the “9 + 2” axoneme arrangement, which consists of nine pairs of doublet microtubules surrounding a central pair. The outer doublets of a motile cilium are linked to dynein arms and radial spokes, which are pivotal in controlling the direction and force of ciliary beating. On the other hand, primary cilia exhibit a “9 + 0” axoneme configuration, lacking the central microtubule pair, dynein arms, and radial spokes, thereby rendering them immotile [16] (Fig. 1).

Other components of the cilium include the ciliary membrane, basal body, and transition zone. The ciliary membrane, which is connected to the plasma membrane, envelops the entire axoneme of the cilium. This membrane is enriched with various signaling receptors and ion channels, including those involved in the Hedgehog pathway and Ca^2+^ channels, enabling the cilium to function as an important signaling hub [4, 23]. The basal body, derived from the mother centriole, reverts back to a centriole during ciliary disassembly preceding cell division [1, 24]. The transition zone, located between the basal body and axoneme, regulates the influx and efflux of liquids and proteins, thus establishing the composition within the cilia [25] (Fig. 1). The collective contribution of these intricate structures and components determines the architecture and performance of cilia in cellular processes.

Motile cilia are designed for dynamic movement, facilitating the generation of directed fluid flows through coordinated activity. In contrast, primary cilia act as sensitive probes, capturing various signals from the environment, and triggering responses that are crucial for regulating cell division, development, gene activity, migration, and overall cell and tissue morphology. Owing to their extensive presence in mammalian organisms and critical role in signaling pathways, the same ciliary gene mutations or abnormal expression has the potential to cause varying manifestations of ciliary abnormalities and inconsistent symptoms of ciliopathies [8, 26, 27]. The variability in symptoms can result from factors such as genetic background, environmental influences, the extent of gene mutation or dysregulation, and the specific cell types or tissues affected. Nonetheless, the specific mechanisms underlying ciliopathies remain elusive, leaving ample scope for discovery in this field.

## IFT: protein translocation machinery in cilia

During the growth of the cilium, the axoneme is assembled by the addition of new axonemal subunits to its distal tip. However, cilia lack the machinery that is necessary for protein synthesis, the site of assembly of the axoneme is far removed from the cell body, where the building materials are synthesized. The cell has solved this problem for the delivery of new axonemal building blocks to the site of axonemal assembly by means of IFT [28, 29]. During IFT, the non-membrane-bound particles are moved along the axonemal doublet microtubules, and beneath the ciliary membrane. The anterograde IFT-B particles moving from the ciliary base to the tip for the proper assembly and maintenance of ciliary axoneme and membrane. At the ciliary tip, the building blocks are released, and IFT-B particles are then transported back by IFT-A to the ciliary base [16]. This IFT process is well conserved and required for the assembly of most cilia and eukaryotic flagella. The movement of these IFT particles are driven by motor proteins, including the anterograde kinesin and the retrograde dynein proteins, to move up and down the cilium [30].

## Classification and characterization of kinesins

**Fig. 2:**
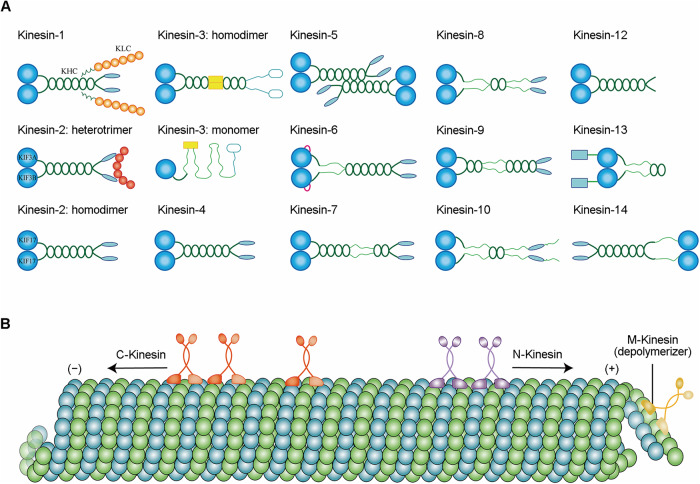
The kinesin superfamily. **A** All kinesins have a motor domain head (see the figure; dark blue), which contributes to ATP hydrolysis for powering the movement along microtubules. Kinesins also contain the neck linker region, tail region and stalk region with coiled-coil segments for oligomerization. Kinesin-1 motors are heterotetramers consisting of the heavy chain KHC and light chain KLC. Kinesin-2 motors have two types: heterotrimers and homodimers. Kinesin-3 motors can also exist as homodimers or monomers. Kinesin-5 motors are heterotetramers. Except for these four kinesins, all other kinesins are homodimers. **B** Kinesins are mainly divided into three types based on the location of their motor domain: C-kinesins, N-kinesins, and M-kinesins. Generally, C-kinesins move towards the minus end of microtubules, N-kinesins move towards the plus end and M-kinesins destabilize microtubules.

Kinesins constitute a superfamily of proteins with 15 members, classified into 14 subclasses (kinesin 1 to kinesin 14B) through phylogenetic analysis [31] (Fig. 2A). Each member of the kinesin superfamily (KIF) possesses a common motor domain, which utilizes the chemical energy from ATP hydrolysis to initiate movement along microtubules. These kinesins are generally divided into three categories based on the location of the motor domain: N-kinesins carry a motor domain in the amino-terminal region, M-kinesins have their motor domain in the middle, and C-kinesins contain it in the carboxy-terminal region. Typically, N-kinesins show directed motility towards the plus (rapidly growing) end of the microtubule, while C-kinesins move towards the minus (slowly growing) end. In contrast, M-kinesins destabilize microtubules instead of migrating along them (Fig. 2B). However, some kinesin-8 (N-kinesin) and kinesin-14 (C-kinesin) motors can both traverse and depolymerize microtubules [32–34]. Furthermore, certain kinesin-5 and kinesin-14 families can cross-link and slide adjacent microtubules, adding complexity to these generalizations [35, 36].

In addition to the motor domain, many kinesins encompass a neck linker region, a stalk region, and a tail domain [37]. The neck linker region, connected to the motor domain, acts as a flexible hinge and assists in transmitting conformational changes during the ATP hydrolysis cycle. The stalk region ensures stability, connecting the motor domain to the cargo-binding tail domain. This tail domain interacts with specific cargo molecules, enabling kinesins to transport various cargoes within cells. Further, coiled-coil segments for oligomerization are present in many kinesins, making most kinesin motors homodimers. For instance, kinesin-1 motors are heterotetramers comprising two subunits: kinesin heavy chain and kinesin light chain; kinesin-2 motors split into two subfamilies, either heterotrimers (KIF3A-KIF3B-kinesin associated protein) or homodimers (KIF17); kinesin-3 motors may exist as monomers or homodimers; and the kinesin-5 family consists of homotetrameric motors (Fig. 2A) [38, 39]. These kinesins, which all belong to the N-kinesins, actively transport cargoes directionally towards the plus-end of microtubules that form cylindrical polymers of 13 protofilaments. Despite the highly conserved nature of their motor domains, their cellular functions vary due to differences in these structural components.

**Fig. 3:**
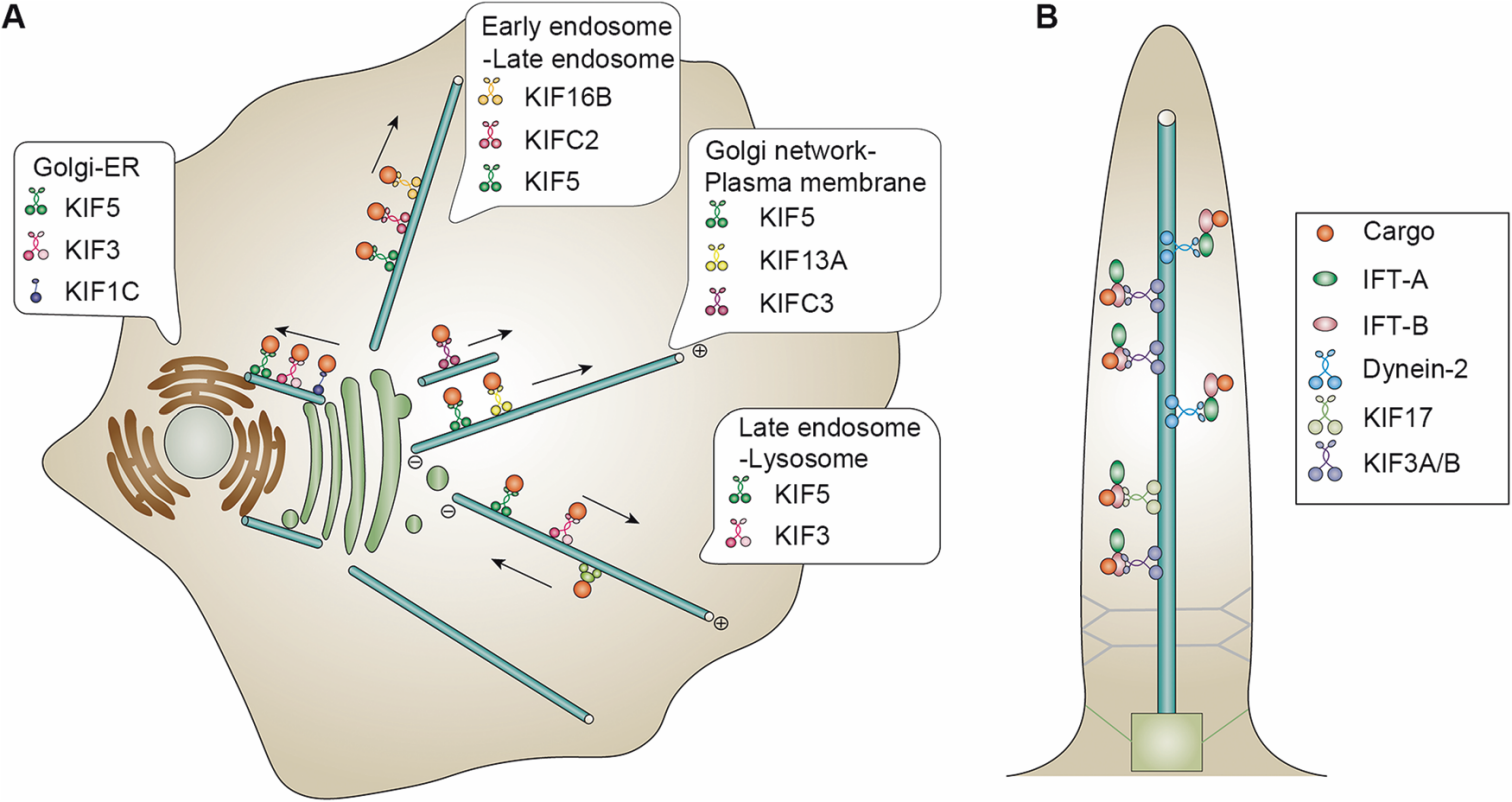
Intracellular transport mediated by kinesins. **A** The process of intracellular transport begins when kinesins bind to vesicles containing organelles or protein complexes, such as endosomes and lysosomes. Kinesins move along microtubules to transport vesicles from the Golgi to the endoplasmic reticulum (ER), as well as from the Golgi network to the plasma membrane. They also facilitate the transportation of lysosomes and endosomes. **B** Kinesin-2 members KIF3A/KIF3B and KIF17 bind IFT-B to transport cargos from the base to the tip of cilia. And then dynein-2 is activated by IFT-A for the retrograde transport.

Kinesins play a plethora of roles in a microtubule-dependent manner. One of their primary functions is vesicle transport, as kinesins assist in the movement of vesicles containing important molecules and organelles to specific locations within cells [39] (Fig. 3A). This transport process is crucial for maintaining cellular functionality and assuring proper distribution of essential components. Another pivotal role of kinesins is macromolecule transport; these proteins aid in the movement of large molecules, such as proteins and nucleic acids, within the cell [40, 41]. By facilitating the transport of these macromolecules, kinesins contribute to key cellular processes like gene expression and cellular signaling. Kinesins also participate in cell division processes tied to mitosis and meiosis, participating in chromosome segregation to ensure accurate distribution of genetic material to daughter cells [42].

It’s noteworthy that kinesins, specifically the known IFT kinesin (kinesin-2), contributes to the dynamic nature of cilia and ensure their proper functioning [43]. By transporting cargoes and signaling molecules and receptors to and from the ciliary membrane, kinesin-2 influences the extension of cilia and the modulation of ciliary signaling pathways [44] (Fig. 3B). Accumulated evidence suggests that a distinctively longer neck linker region in the kinesin-2, which includes an additional three amino acid residues (Asp-Ala-Leu, DAL) at the C-terminus prior to helix α7 [45]. This characteristic underpins the mechanistic foundation for its shorter run lengths, a trait that seems to be adapted to its specific role of transporting ciliary proteins along the axoneme of cilia.

Moreover, recent works have underscored the significant involvement of non-IFT kinesins in the assembly and maintenance of cilia. While the mechanisms through which non-kinesins contribute to ciliary homeostasis remain an active area of research, preliminary findings suggest that these kinesins may be involved in various ciliary processes, such as the regulation of ciliary length, the transport of specific cargoes, or the modulation of ciliary signaling pathways. Therefore, further research into the roles of these non-IFT kinesins may yield new insights into the molecular mechanisms underlying ciliary function and dysfunction, and ultimately lead to the development of novel therapeutic strategies for ciliopathies.

## IFT kinesins

There are two types of anterograde IFT motors, namely the heterotrimeric kinesin-2 and the homodimeric OSM-3 or KIF17. These kinesins belong to the kinesin-2 family, also known as IFT kinesins. The heterotrimeric kinesin-2 complex consists of KIF3A/KIF3B/KIFAP3 and can move at a rate of 0.2-2.4 μm/s, depending on the species and ciliary type [46]. On the other hand, OSM-3 or KIF17 acts as a homodimer and moves approximately 1.3 μm/s along the ciliary axoneme [47, 48] (Fig. 3B). The biogenesis of cilia requires the anterograde IFT driven by kinesin-2, as it is responsible for transporting IFT trains. These trains are believed to deliver axoneme precursors to the tip of the axoneme, where they are incorporated, and to organize and move ciliary membrane-associated signaling complexes. For example, in the green alga *Chlamydomonas*, inactivation of the FLA10 subunit of heterotrimeric kinesin-2 using conditional mutants leads to a gradual halt in IFT and defects in the assembly or maintenance of motile cilia [49]. This observation supports the hypothesis that heterotrimeric kinesin-2 drives the anterograde transport of IFT trains.

The role of kinesin-2 motors in the assembly of sensory cilia in *Caenorhabditis elegans* amphid channels differs and presents a more intricate process [50]. The axonemes of these cilia possess a bipartite structure characterized by a core comprising nine doublet microtubules known as the middle segment. From this middle segment, nine singlet microtubules extend to form the distal segment, which plays a critical role in certain forms of chemosensory signaling. The assembly of these axonemes involves a unique and unexpected collaboration between the heterotrimeric kinesin-2, kinesin-II, and the homodimeric kinesin-2, OSM-3. In this collaboration, the middle-segment assembly involves both motors transporting IFT trains along the middle segment, while the distal-segment assembly depends only on OSM-3 transporting IFT trains along the distal segment. Therefore, in wild-type animals, kinesin-II and OSM-3 both contribute redundantly to the assembly of the middle segment, while OSM-3 alone is responsible for constructing the distal segment.

In contrast, the cilia found on olfactory receptor neurons in *Drosophila* also exhibit a bipartite organization and develop through a different two-step pathway [51]. However, in this case, heterotrimeric kinesin-2 alone appears to be sufficient for the assembly of the entire axoneme. In mice, heterotrimeric kinesin-2 may have additional ciliogenic functions beyond driving IFT that cannot be compensated for KIF17, as it is required for the proper organization of centrioles, which form the basal body of the cilium [52]. Additionally, in zebrafish, the absence of KIF17 results in a loss or disorganization of outer segments in retinal photoreceptors, while it does not affect the formation of motile cilia in the pronephros [53]. These observations indicate that diverse mechanisms for employing kinesin-2 motors have evolved to facilitate cilium assembly.

## Non-IFT kinesins

**Fig. 4:**
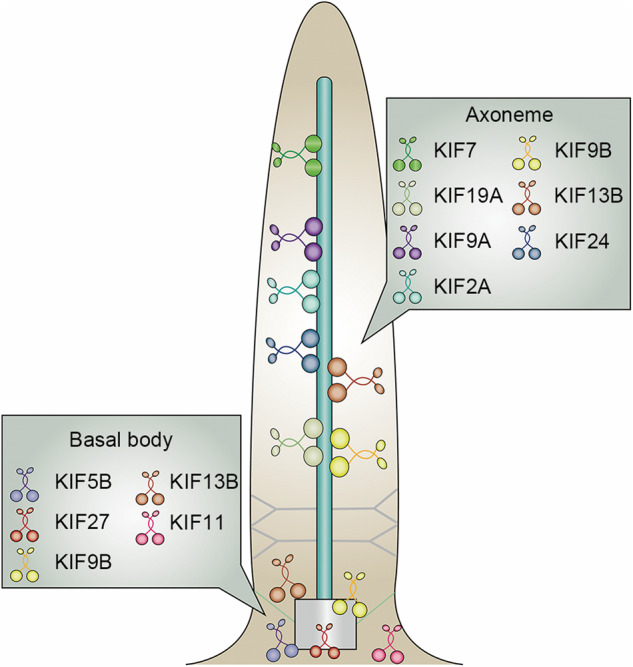
Non-IFT kinesins in cilia. Non-IFT kinesins are distributed at various strategic locations within cilia, such as the axoneme and basal body. The distinct compartments of non-IFT kinesins highlight their diverse roles in the regulation of ciliary structure and function.

Beyond the well-known IFT kinesins, recent works have unveiled the involvement of non-IFT kinesins in ciliary homeostasis maintenance. These kinesins were found located at the basal body or axoneme of cilia and contribute to regulate ciliary length and the ciliary signaling pathways (Fig. 4). Such roles could be expected for the members of the kinesin-13 and kinesin-4 subfamily, known to have microtubule depolymerizing activities, therefore, negatively controlling the length of axonemal microtubules and the ciliary dependent Hedgehog signaling pathway [20, 21, 54]. In addition to the depolymerizing kinesins, knockout of certain kinesin genes has identified several new kinesin members involved in diverse function at cilia.

### Kinesin-1 (KIF5B)

Kinesin-1, the first identified plus-end-directed microtubule motor, is involved in various cellular processes through its interactions with different cargoes such as vesicles, organelles, mRNAs, and multiprotein complexes [55, 56]. Kinesin-1 is a heterotetramer composed of two heavy chains and two light chains. The microtubule binding motor region is found in the N-terminus of the heavy chain, which can be encoded by three different genes (*Kif5A, Kif5B, Kif5C*). KIF5A and KIF5C are expressed exclusively in neurons, while KIF5B is ubiquitous. Each heavy chain dimer associates with two copies of KLC1 or KLC2, which are expressed in most cell types [39].

Studies have indicated that KIF5B and KLC1 localized to the basal body and play an inhibitory role in ciliary extension, as depletion of these proteins leads to abnormally elongated cilia. Knockdown of KIF5C alone does not significantly affect ciliary length, and KIF5A is not highly expressed in hTERT-RPE cells, a cell line known to induce ciliary formation in vitro. Furthermore, genetic interaction studies suggest that the nuclear/cytoplasmic distribution of CCDC28B, a protein associated with Bardet-Biedl syndrome, is influenced by KIF5B, as targeting KIF5B leads to nuclear accumulation of CCDC28B [57].

### Kinesin-3 (KIF13B/KLP-6)

Kinesin-3 family members are plus-end directed motors involved in vesicle transport and endocytosis. Among them, KIF13B (also known as guanylate kinase-associated kinesin or GAKIN) is implicated in the regulation of neuronal polarity, axon formation and myelination, Golgi to plasma membrane trafficking, germ cell migration, and planar cell polarity signaling [39].

Recent studies have shown that KIF13B undergoes bursts of IFT-like bidirectional movement within primary cilia, and its depletion leads to ciliary accumulation of the cholesterol-binding membrane protein CAV1 and impaired Hedgehog signaling [58, 59]. Additionally, the velocities of anterograde and retrograde intraciliary movement of KIF13B are similar to those of IFT, but its movement within the cilium requires its own motor domain. Interestingly, the homolog of KIF13B, KLP-6, has been observed to move in cilia of *Caenorhabditis elegans* and modulate the velocities of IFT and kinesin-2 motors. KLP-6 acts as a positive regulator of ciliary length extension, as its accumulation in the cephalic male cilia promotes elongation of cilia [60]. This demonstrates the modulation of general kinesin-2-driven IFT processes by kinesin-3 in the cilia of *Caenorhabditis elegans* male neurons.

### Kinesin-4 (KIF7/KIF27)

Kinesin-4 is a remarkable motor protein due to its unique ability to depolymerize microtubules [61]. It plays critical roles in cell division, microtubule organization, and signal transduction. Among its members, KIF7 serves as a conserved regulator of the Hedgehog signaling pathway. This kinesin facilitates the transmission of signals from the membrane protein Smoothened to the Gli/Gi transcription factors. A recent finding suggests that KIF7 regulates the length of the microtubule plus end and promotes the precise localization and proper regulation of Gli and the inhibitory factor Sufu at the tip of primary cilia. Furthermore, KIF7 mutations cause primary cilia abnormalities, including excessive length, twisting, and instability. These defects lead to the formation of ectopic tip-like compartments where Gli-Sufu complexes become localized and inappropriately activated in the absence of the sonic hedgehog ligand [21].

Another member of the kinesin-4 family, KIF27, also plays a role in cilia-related processes. KIF27, the closest mammalian homologue of KIF7, is found in motile cilia and share the ability of KIF7 to regulate axonemal microtubule dynamics. Specifically, KIF27 contributes to the assembly of the central pair of microtubules in “9 + 2” motile cilia through its interaction with Fused [62]. Mice with defective KIF27 exhibit suppurative inflammatory responses in the nasal passages and middle ear, as well as hydrocephalus [63].

### Kinesin-5 (KIF11)

Kinesin-5, also known as kinesin family member 11 (KIF11) or Eg5, plays crucial roles in the formation and maintenance of bipolar spindle orientation during cell division. These activities are facilitated by its unique antiparallel tetrameric structure, which enables the motor protein to crosslink and slide adjacent microtubules [64]. Apart from its mitotic functions, KIF11 has also been found to have non-mitotic roles, including protein transport from the Golgi complex to the cell surface, regulation of axonal growth and branching, and ciliary formation [17, 18, 65, 66].

Our previous research has shown that KIF11 localizes to the basal body of primary cilia in various cell types. Knockdown of KIF11 expression in RPE1 cells leads to a decrease in ciliary length and number and perturbs Hedgehog signaling [17]. Another study further supports the non-mitotic role of KIF11 in cilia, demonstrating that KIF11 plays a critical role in regulating ciliary behavior [18]. Moreover, KIF11 expression is significantly higher in glioblastoma cells compared to normal cells, and there is also an overexpression of Hedgehog signaling in glioblastoma [67]. These suggest that KIF11-mediated ciliogenesis may contribute to the overactivation of Hedgehog signaling in glioblastoma cancer cells, which holds potential implications for future cancer treatment strategies.

### Kinesin-8 (KIF19A)

Kinesin-8 members possess remarkable capabilities of both walking towards the plus-ends of microtubules and depolymerizing these ends upon arrival, thereby exerting control over microtubule length [33]. These motor proteins are observed on cytoplasmic microtubules during interphase and near kinetochores during cell division. Disruption of their function during mitosis leads to the formation of excessively long spindle microtubules, resulting in aberrant chromosomal segregation. This observation strongly supports the notion that precise regulation of microtubule length by kinesin-8 motors is crucial for accurate cell division.

Among these motors, KIF19A has been extensively studied for its role in regulating ciliary length by depolymerizing microtubules at the tips of cilia. Depletion of KIF19A in mice results in the manifestation of ciliopathy phenotypes, including hydrocephalus and female infertility, caused by the presence of abnormally elongated cilia that are unable to generate proper fluid flow [68]. Recent research has indirectly demonstrated that KIF19A plays a pivotal role in mediating ciliary length in mammals. For instance, depletion of adenylate cyclase 6 in mice leads to elongated cilia in airway epithelial cells, primarily due to decreased KIF19A protein levels in the cilia resulting from its degradation through autophagy [69]. These studies shed light not only on the genetic regulation of cilia by KIF19A but also on the mechanisms underlying the regulation or control of KIF19A itself.

### Kinesin-9 (KIF9A/KIF9B)

Kinesin-9 members are motor proteins that are exclusively expressed in tissues containing motile cilia or flagella, such as the testis, brain, and lung, as well as in flagellated microorganisms like *Giardia, Leishmania, and Chlamydomonas*. These kinesin motors primarily move towards the plus end of microtubules. The kinesin-9 family consists of two subfamilies: KIF9A, which includes *Chlamydomonas reinhardtii* KLP1, and KIF9B, which includes human KIF6. KLP1 is localized to the central pair microtubules of the axoneme and plays a role in influencing flagellar motility [70]. Disruption in KLP1 function leads to flagella that beat slowly or become paralyzed.

Recent studies have highlighted the importance of KIF9 in ciliary motility. KIF9 is highly conserved across evolutionary species and is considered the vertebrate ortholog of KLP1. It has been reported that KIF9 localizes to the axoneme of sperm flagella and cilia in multiciliated cells, such as those found in *Xenopus* and human airways. KIF9 is responsible for maintaining proper ciliary motility and the integrity of the distal end of the axoneme [19]. In contrast, KIF6 is localized to both the axoneme and basal body of multiciliated cells. It is not only essential for ciliary motility but also plays a specific role in the formation of cilia in ependymal cells. Studies have shown that mutations in *Kif6* can lead to neurodevelopmental defects and intellectual disability in humans [71].

### Kinesin-13 (KIF24/KIF2A)

The kinesin-13 family specifically contains M-kinesins. Unlike conventional kinesins, kinesin-13 proteins do not walk along microtubules but instead depolymerize them using ATP. This depolymerizing activity of kinesin-13 proteins operates in a range of physiological contexts such as spindle assembly, chromosome segregation, and axonal growth.

Early studies have shown that kinesin-13 members in *Giardia, Leishmania, and Chlamydomonas* are localized to axonemes and play a role in regulating the length of flagellar [72–74]. However, in the mammalians, the kinesin-13 members consist of KIF2A, KIF2B, KIF2C/MACK, and KIF24. KIF24 has been reported to block ciliogenesis by recruiting CP110 at the mother centrioles and remodeling centriole microtubules through its microtubule-depolymerizing activity [24, 54]. Moreover, research has demonstrated that even in cycling cells, knockdown of KIF24 by small interfering RNA leads to inappropriate ciliogenesis. Another kinesin-13 member, KIF2A, has been shown to have the ability to disassemble primary cilia by depolymerizing microtubules in response to growth signals, with its activity controlled by the PLK1 [75].

## Emerging roles of non-IFT kinesins in ciliopathies

Considering the pivotal contribution of non-IFT kinesins to the maintenance of ciliary homeostasis, it is unsurprising that these kinesin motors are intricately linked with a spectrum of ciliopathies. Microcephaly, a neurological malformation that characterized by an abnormal small head circumference, is one of the most frequently associated clinical signs [76]. Notably, mutations in the genes encoding kinesin motors-KIF1B, KIF14, KIF16B, KIF11, KIF10, KIF15, and KIF2A-have been identified in numerous patients with microcephaly [77–79].

Non-IFT kinesins are also involved in other neuronal disorders related to ciliopathies. For instance, *KIF4A*, *KIF6* and *KIF7* has ascended to prominence as a putative gene of interest in the etiology of hydrocephalus [80–82]. Investigations into the developmental biology of KIF26A underscore its potential role in neural system development, as knockout mice models reveal critical deficits such as enteric nerve hypoplasia [83, 84]. The proteins KIF1A and KIF5 are of paramount importance for higher-order brain functionalities, namely learning and memory, exerting influence through the modulation of synaptic transmission [85]. Peripheral neuropathies represent yet another sphere in which KIF1A and KIF1B demonstrate a genetic association [86].

Transgenic models, particularly mice with targeted deletions of KIF genes, have surfaced with a spectrum of ciliopathy syndromes. These include kidney disorders resultant from KIF26B mutations [87], and KIF19A depletion leading to female infertility [22]. Complementing these insights, recent discoveries have delineated biallelic variants of *KIF24* as pathogenic factors in skeletal ciliopathies, encompassing variants such as acromesomelic skeletal dysplasia and spondylometaphyseal dysplasia [88]. Furthermore, genetic variants in *KIF1B*, *KIF21B*, and *KIF5A* have been associated with increased vulnerability to multiple sclerosis [89–91]. Collectively, these evidences reinforce the notion that non-IFT kinesins are crucial to ciliary function and, when impaired, to the pathogenesis of a multitude of abnormalities related to ciliopathies.

## Concluding remarks

The study of kinesins and their roles in cilia biology has undergone significant advancements over recent years, revealing the intricate mechanisms by which these motor proteins contribute to ciliary assembly, maintenance, and function. In this review, we have discussed the emerging roles of non-IFT kinesins in cilia-related processes, providing insights into their diverse functions and their implications for cellular homeostasis and human health. While IFT kinesins have long been recognized as central players in cilia assembly and maintenance, the discovery of non-IFT kinesins’ involvement adds a layer of complexity to our understanding of ciliary activities. Emerging evidence compellingly indicates that the various kinesin families are interdependent, collaboratively maintaining ciliary homeostasis. The observed interplay between IFT-associated and non-IFT kinesin proteins poses fascinating questions regarding their mechanisms of communication and cooperation. This teamwork is crucial for the modulation of the ciliary length, the precision of cargo transport, and the nuanced modulation of signaling pathways. Future research aimed at deciphering the crosstalk between these kinesin families will provide deeper insights into the mechanisms governing cilia biology.

The identification of non-IFT kinesins as critical players in cilia-related processes has important implications for our understanding of ciliopathies. Mutations in various ciliary components, including kinesins, have been linked to the development of ciliopathies, underscoring the significance of these motor proteins in maintaining cellular homeostasis [8, 13]. For example, the presence of KIF11 in the connecting cilium of photoreceptors, and the identification of *KIF11* mutations in patients with retinal diseases such as MLCRD (microcephaly, lymphedema, and chorioretinal dysplasia), CDMMR (chorioretinal dysplasia, microcephaly, and mental retardation), and FEVR (familial exudative vitreoretinopathy) suggest that KIF11 may play a vital role in the pathological processes of these conditions by mediating photoreceptor ciliary homeostasis [79, 92, 93]. Elucidating the roles of non-IFT kinesins in cilia biology may offer valuable insights into the molecular mechanisms underlying ciliopathy pathogenesis. Furthermore, as multiple members of the kinesin family are continuously being identified as potential targets for treating various diseases, including cancer [94], exploring cilia and ciliary proteins as a strategy for addressing ciliopathies holds great promise [95, 96].

As the field of kinesin research continues to advance, several intriguing questions and avenues for future investigation arise. For example, ciliary homeostasis represents a complex and finely tuned regulatory process encompassing assembly, disassembly, and maintenance phases [97], yet the specific contributions of different kinesin proteins within this balance are not well understood. Moreover, the mechanisms by which multiple members of this large kinesin family work in concert remain elusive. Most importantly, the physiological and pathological significance of kinesin-mediated ciliary homeostasis in development and human disease remains unclear. These knowledge gaps present a compelling case for future research to unravel the intricate orchestra of kinesin activities that maintain ciliary homeostasis and to decipher their broader implications in health and disease.

The core IFT-dependent machinery is crucial for the transport of ciliary and signaling proteins. However, in certain ciliated protists that are devoid of genes encoding for IFT components, and in conjunction with some metazoan spermatozoa, they use IFT-independent mechanisms for assembling axonemes that exposed to the cytosol [98, 99]. During this process, all or portion of this axoneme, at least temporarily, is not enveloped by plasma membrane but is instead exposed to the cytoplasm. This distinct IFT-independent ciliogenesis pathway permits a robust exchange with cytosolic proteins, and consequently, IFT is presumably excluded from playing any direct part in such cytosolic ciliogenesis events. This unconventional pathway delivers profound insights into the molecular machinations that govern non-IFT kinesins in maintaining ciliary homeostasis. For example, the basal body-localized KIF11, a newly-identified pivotal protein in ciliogenesis, which strikingly lacks inherent motor activity but is vitally influential in ciliary length. To date, the molecular mechanisms underpinning the role of KIF11 are unclear. With this context, we postulate that KIF11 may harness a mechanism resonant with the IFT-independent ciliogenesis pathway. Such a role would likely entail exploiting the cytoplasm’s microtubule framework to effectuate the translocation and assembly of requisite constituents for ciliary construction, providing a greater understanding of this kinesin protein’s involvement in the complex narrative of cilia formation and maintenance.

The development of advanced imaging techniques will enable researchers to visualize kinesin behavior within cilia with unprecedented detail, offering new insights into their functions. Continued functional studies in model organisms and human genetics will provide valuable information about the roles of non-IFT kinesins in various biological contexts. In conclusion, the emerging roles of non-IFT kinesins in cilia biology have broadened our understanding of ciliary dynamics and cellular function. These motor proteins contribute to a range of processes within cilia, including assembly, length regulation, cargo transport, and signaling. By shedding light on the specific ciliary activities of non-IFT kinesins, their implications for ciliopathies, and their diverse functions beyond cilia, this review emphasizes the intricate and multifaceted nature of kinesin-mediated regulation in cellular processes. As research in this field progresses, we anticipate that further insights into the roles and mechanisms of non-IFT kinesins will continue to shape our understanding of cellular biology and human health.

## Data Availability

Data sharing is not applicable, as no datasets were generated or analyzed during this study.
